# Structural Diversity of Sense and Antisense RNA Hexanucleotide Repeats Associated with ALS and FTLD

**DOI:** 10.3390/molecules25030525

**Published:** 2020-01-25

**Authors:** Tim Božič, Matja Zalar, Boris Rogelj, Janez Plavec, Primož Šket

**Affiliations:** 1 Slovenian NMR Centre, National Institute of Chemistry, Hajdrihova 19, SI-1000 Ljubljana, Slovenia; timbozicbiochemistry@gmail.com (T.B.); m.zalar@beatson.gla.ac.uk (M.Z.); 2 Department of Biotechnology, Jožef Stefan Institute, Jamova 39, SI-1000 Ljubljana, Slovenia; boris.rogelj@ijs.si; 3 Biomedical Research Institute BRIS, Puhova 10, SI-1000 Ljubljana, Slovenia; 4 EN-FIST Center of Excellence, Trg OF 13, SI-1000 Ljubljana, Slovenia; 5 Faculty of Chemistry and Chemical Technology, University of Ljubljana, Večna pot 113, SI-1000 Ljubljana, Slovenia

**Keywords:** NMR, RNA, ALS, FTLD, *C9orf72*

## Abstract

The hexanucleotide expansion GGGGCC located in *C9orf72* gene represents the most common genetic cause of amyotrophic lateral sclerosis (ALS) and frontotemporal lobar dementia (FTLD). Since the discovery one of the non-exclusive mechanisms of expanded hexanucleotide G_4_C_2_ repeats involved in ALS and FTLD is RNA toxicity, which involves accumulation of pathological sense and antisense RNA transcripts. Formed RNA foci sequester RNA-binding proteins, causing their mislocalization and, thus, diminishing their biological function. Therefore, structures adopted by pathological RNA transcripts could have a key role in pathogenesis of ALS and FTLD. Utilizing NMR spectroscopy and complementary methods, we examined structures adopted by both guanine-rich sense and cytosine-rich antisense RNA oligonucleotides with four hexanucleotide repeats. While both oligonucleotides tend to form dimers and hairpins, the equilibrium of these structures differs with antisense oligonucleotide being more sensitive to changes in pH and sense oligonucleotide to temperature. In the presence of K^+^ ions, guanine-rich sense RNA oligonucleotide also adopts secondary structures called G-quadruplexes. Here, we also observed, for the first time, that antisense RNA oligonucleotide forms i-motifs under specific conditions. Moreover, simultaneous presence of sense and antisense RNA oligonucleotides promotes formation of heterodimer. Studied structural diversity of sense and antisense RNA transcripts not only further depicts the complex nature of neurodegenerative diseases but also reveals potential targets for drug design in treatment of ALS and FTLD.

Academic Editor: 

## 1. Introduction

The pathological hexanucleotide expansion GGGGCC located in the first intron or promoter region of the *C9orf72* gene on chromosome 9p21 represents the most common genetic cause of amyotrophic lateral sclerosis (ALS) and frontotemporal lobar dementia (FTLD)—two fatal neurodegenerative diseases with progressive loss of motor neurons in the brain and spinal cord [1,2,3,4,5,6,7]. Healthy individuals possess up to 19 repeats, while patients with ALS or FTLD may carry up to 5000 repeats [3,4,5,6]. To date, three main non-exclusive mechanisms of expanded hexanucleotide G_4_C_2_ repeats involved in ALS and FTLD have been proposed: protein C9orf72 haploinsufficiency, accumulation of dipeptide repeat proteins (DPRs) and RNA toxicity [7]. While haploinsufficiency and DPRs are both protein driven mechanisms, RNA toxicity involves accumulating sense and antisense RNA transcripts. Accumulation leads to formation of RNA foci, which sequesters RNA-binding proteins [8]. Bound RNA-binding proteins are responsible for nuclear transport, splicing, and translation, all of which are affected by dysregulated RNA metabolism [9,10]. Myotonic dystrophy and fragile X-associated tremor/ataxia syndrome are also repeat expansion disorders, where RNA toxicity plays a crucial role in pathogenesis [11,12]. This indicates that structures and structural motifs adopted by different repeat expansions direct the final outcome as they dictate the binding affinity and specificity of protein domains. Latter hypothesis led to several structural studies of DNA and RNA repeat expansion variations of *C9orf72* gene [1,2,10,13,14,15,16,17,18]. While G-rich sense RNA repeat expansion variations seem to form mainly parallel G-quadruplexes, C-rich antisense RNA repeat expansion is presumably limited to hairpins and A-form-like dimer with regularly spaced tandem C:C mismatches [16,17,18]. Although i-motifs have been proposed for antisense DNA repeat expansion variations [1], they have not been reported yet for antisense RNA repeat expansion, to the best of our knowledge.

By utilizing NMR spectroscopy and complementary methods we demonstrated that sense r(G_4_C_2_)_4_ and antisense r(G_2_C_4_)_4_ RNA oligonucleotides composed of four hexanucleotide repeats can form dimers and hairpins under conditions approaching physiological relevance. While structures of antisense RNA oligonucleotide show pH dependency, structures of sense RNA oligonucleotide are temperature sensitive. Furthermore, sense RNA oligonucleotide forms structurally different G-quadruplexes in the presence of KCl and antisense RNA oligonucleotide indicates i-motif formation at low pH and temperature. Simultaneous presence of both RNA oligonucleotides revealed formation of heterodimer although structures adopted by individual RNA oligonucleotides seem to persist but are a minority.

## 2. Results

### 2.1. Homodimer–Hairpin Equilibrium of r(G_2_C_4_)_4_ is pH-Dependent

**Figure 1:**
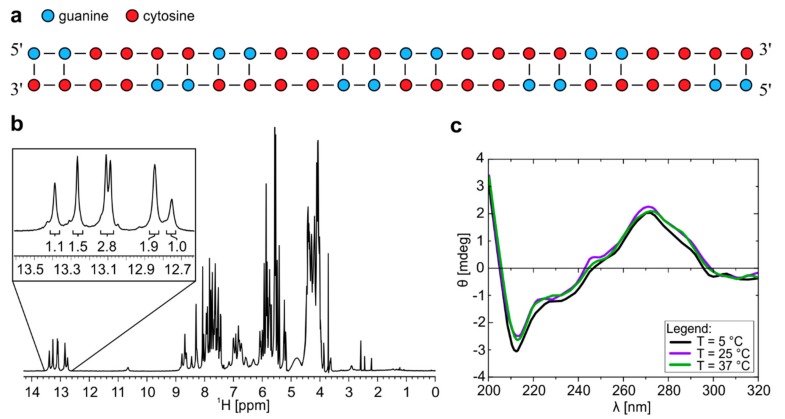
^1^H-NMR and CD spectra of r(G_2_C_4_)_4_ at pH 7.0 forms dimeric structure. (**a**) Schematic presentation of homodimer with G-C base pairs and C-C mismatches. (**b**) ^1^H-NMR spectrum of r(G_2_C_4_)_4_ in 10% ^2^H_2_O at pH 7.0, 25 °C and 0.9 mM oligonucleotide concentration per strand. Numbers below the signals represent integral values. **c**) CD spectra of homodimer with concentration of 100 µM per strand at 5, 25 and 37 °C.

Analysis of NMR spectra of r(G_2_C_4_)_4_ allowed us to propose secondary structure of homodimer consisting of G-C base pairs and C-C mismatches adopted at pH 7.0 (Figure 1a). Dimeric nature of structure was identified based on translational diffusion coefficient (D_t_) value of 0.91 ± 0.05 × 10^−10^ m^2^s^−1^. 1D ^1^H-NMR spectrum reveals six well resolved signals between δ 12.6 and 13.6 ppm corresponding to guanine imino protons involved in Watson-Crick base pairs (Figure 1b and Figure S1a). Relative integral ratio of signals suggests that homodimer consists of 16 G-C base pairs. Predominant formation of a dimeric symmetric structure is in agreement with observation of 16 cross-peaks corresponding to cytosine H5/H6 connectivities in 2D TOCSY NMR spectrum of the studied oligonucleotide with 16 cytosine residues (Figure S2). Examination of imino regions of ^1^H-NMR and CD spectra, as well as UV melting experiment on r(G_2_C_4_)_4_ at pH 7.0 showed that homodimer is thermally stable in temperature range from 5 to 70 °C (Figure 1c, Figures S3 and S4). In oligonucleotide concentration range from 2.6 to 9.3 µM per strand, temperatures of half transition obtained from melting curves during unfolding were between 73 and 76 °C. At 20 µM oligonucleotide concentration, homodimer was not completely unfolded even at 90 °C.

CD spectra of r(G_2_C_4_)_4_ at pH 7.0 reveal minimum at wavelength of 212 nm, maxima at 245 and 265 nm as well as shoulder at 285 nm (Figure 1c), which is in good agreement with CD spectra of A-form dsRNA and r((C_4_G_2_)_n_CCCC) repeats [18,19]. A shoulder in CD spectra at 285 nm, however, suggests the presence of hairpin structure in lesser extent, which is not observable by NMR [20].

^1^H-NMR spectra of r(G_2_C_4_)_4_ acquired at 25 °C as a function of pH ranging between 7.0 and 4.5 show appearance of signals, which indicate shift in equilibrium towards formation of a new structure upon lowering pH (Figure S5). At pH 4.5 signals between δ 12.5 and 14.0 ppm correspond to guanine imino protons involved in Watson-Crick G-C base pairs, while signal with chemical shift of δ 16.45 ppm corresponds to imino protons of hemi-protonated C^+^-C base pairs (Figure S1b). At pH 4.5 the measured value of D_t_ of 1.40 ± 0.05 × 10^−10^ m^2^s^−1^ is in accordance with formation of a monomeric structure (Table S1). Using coordinates from 3D models of homodimer and hairpin of r(G_2_C_4_)_4_ predicted by MC-Fold/MC-Sym enabled to calculate D_t_ with HydroPro program [21]. Comparison of experimental and calculated D_t_ values unambiguously confirmed formation of hairpin and homodimer at pH 4.5 and 7.0, respectively. Homodimer–hairpin equilibrium was also observed in the presence of 10% *w*/*v* PEG, however, imino signal corresponding to hemi-protonated C^+^-C base pairs at δ 16.45 ppm was not observed even at pH 4.5 (Figure S6).

**Figure 2:**
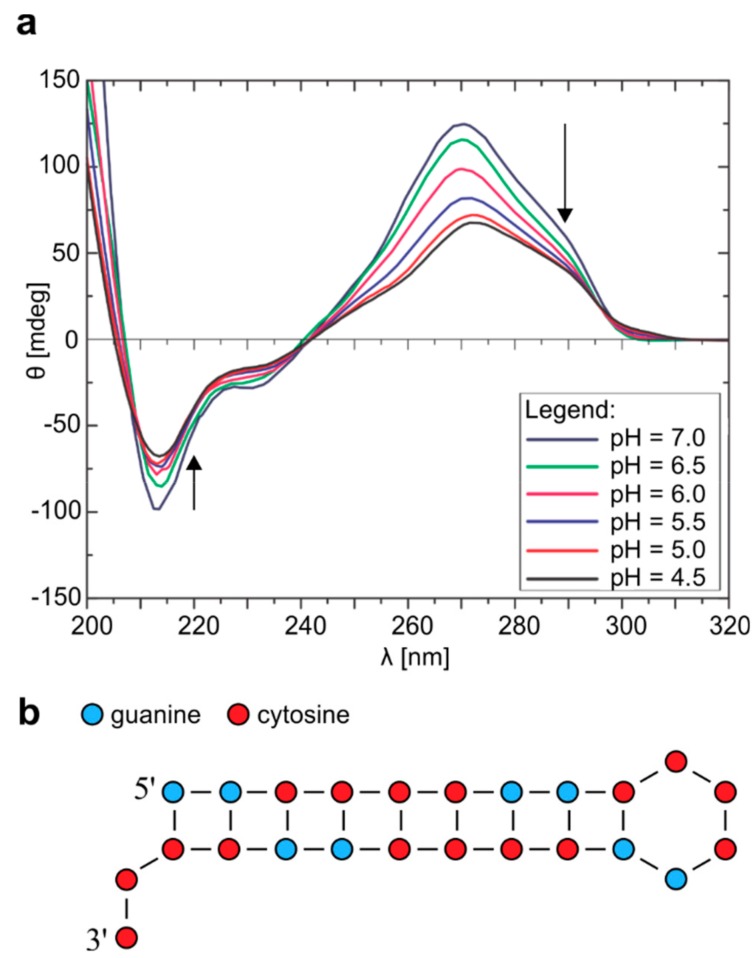
CD spectra of r(G_2_C_4_)_4_ and proposed hairpin adopted at pH 4.5. (**a**) CD spectra of r(G_2_C_4_)_4_ with pH ranging from 7.0 to 4.5. All CD spectra were acquired at 25 °C and concentration of 100 µM per strand. Arrows represent the direction of change in pH. (**b**) Schematic presentation of hairpin formed by r(G_2_C_4_)_4_ at pH 4.5 and 25 °C.

Structural changes caused by variation of pH were also indicated in CD spectra acquired at 25 °C where lowering of pH to 4.5 resulted in less distinct negative band at 212 nm, shift of positive band from 270 nm to 267 nm, while shoulder at 285 nm became more prominent (Figure 2a). Overall, signals in CD spectrum at pH 4.5 were attributed to a hairpin like structure. Both ^1^H-NMR and CD spectra after increasing of pH back to 7.0 show that the process is reversible (Figure S7).

Homodimer–hairpin equilibrium was confirmed by 20% native PAGE at pH 6.0 and 5 °C, where two bands were observed (Figure S8). One band migrating as approximately 30 bp construct corresponds to homodimer, while the other band observed just below 15 bp corresponds to a hairpin structure.

NOE contact between signal at δ 16.45 ppm and signal corresponding to imino protons of guanines involved in Watson-Crick G-C base pairs at δ 13.18 ppm in 2D NOESY spectrum acquired at pH 4.5, 5 °C and mixing time of 100 ms is in agreement with formation of hairpin structure consisting of hemi-protonated C^+^-C base pairs (Figure S9). Imino-imino NOE contacts between three signals resonating from δ 13.0 to 13.3 ppm and signal at δ 13.87 ppm indicate that the latter signal corresponds to imino protons of guanine residues involved in G-C base pairs adjacent to G-C base pairs next to C^+^-C base pairs (Figure 2b).

^1^H-NMR spectra at pH 4.5 as well as UV melting experiment on r(G_2_C_4_)_4_ at pH 5.0 show that hairpin is thermally stable in temperature range between 5 and 70 °C (Figures S10 and S11). In oligonucleotide concentration range from 2.6 to 20 µM the temperature of half transition of hairpin adopted by r(G_2_C_4_)_4_ was estimated to be approximately 90 °C as completely unfolded state was not reached.

At a temperature of 5 °C and pH 4.5 imino region of ^1^H-NMR spectrum revealed also a signal of low intensity at δ 15.75 ppm corresponding to additional hemi-protonated C^+^-C base pairs (Figure S10). According to the chemical shift value, these hemi-protonated C^+^-C base pairs are part of i-motif structure.

### 2.2. Temperature Changes Affect Population of Structures Formed by r(G_4_C_2_)_4_

**Figure 3:**
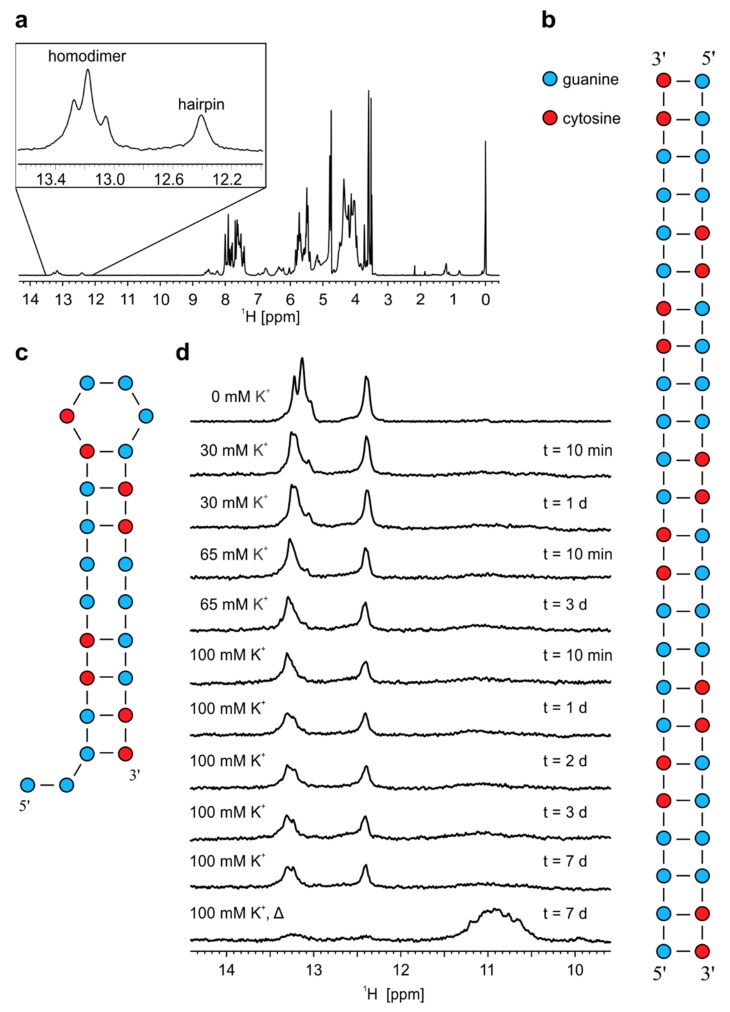
^1^H-NMR spectra and proposed models of structures adopted by r(G_4_C_2_)_4_. (**a**) ^1^H-NMR spectrum of r(G_4_C_2_)_4_ in 10% ^2^H_2_O at pH 6.0, 25 °C and 0.9 mM oligonucleotide concentration per strand. Schematic presentation of (**b**) homodimer and (**c**) hairpin adopted by r(G_4_C_2_)_4_. (**d**) Imino regions of ^1^H-NMR spectra of r(G_4_C_2_)_4_ in 10% ^2^H_2_O at different concentrations of K^+^ ions. KCl solution was gradually titrated into the sample up to the stated concentration of K^+^ ions, displayed on the left of the NMR spectra. Δ represents NMR spectrum after annealing while t on the right represents time after each addition of K^+^ ions until acquisition of NMR spectrum. All spectra were acquired at 25 °C, pH 6.0 and 0.2 mM oligonucleotide concentration per strand.

1D ^1^H-NMR spectrum of r(G_4_C_2_)_4_ recorded at pH 6.0 and 25 °C reveals four well resolved signals between δ 12 and 14 ppm corresponding to guanine imino protons of Watson-Crick base pairs (Figure 3a). 2D DOSY experiment resulted in two sets of aromatic and imino signals among which three imino signals between δ 13.0 and δ 13.4 ppm with D_t_ value of 0.94 ± 0.05 × 10^−10^ m^2^s^−1^ correspond to G-C base pairs of homodimer, while signal resonating at δ 12.41 ppm with D_t_ value of 1.40 ± 0.05 × 10^−10^ m^2^s^−1^ corresponds to G-C base pairs of hairpin. Noteworthy, experimentally observed Dt values are in agreement with values calculated for the proposed models of homodimer and hairpin using HydroPro program (*vide supra*).

Decrease of temperature to 0 °C results in disappearance of imino signal at δ 12.41 ppm corresponding to G-C base pairs of hairpin, while three signals between δ 13.0 and 13.4 ppm corresponding to G-C base pairs of homodimer are still present (Figure S12). Additionally, a new set of imino signals between δ 10.5 and 11.0 ppm starts to appear, which correspond to N1-carbonyl G-G base pairs stabilized at lower temperatures within homodimer (Figure S1c). Integration of imino signals in ^1^H-NMR spectrum of r(G_4_C_2_)_4_ at pH 6.0 and 0 °C shows that the ratio between G-C and G-G base pairs of homodimer is 2:1, which suggests that homodimer of r(G_4_C_2_)_4_ consists of 16 G-C base pairs and eight G-G base pairs (Figure S13 and Figure 3b). On the other hand, most favourable secondary structure of hairpin adopted by r(G_4_C_2_)_4_ with the highest amount of G-C base pairs is proposed in Figure 3c.

^1^H-NMR spectra at different pH values show that homodimer and hairpin of r(G_4_C_2_)_4_ are stable within narrow pH range from 5.0 to 6.0 (Figure S14). At pH 7.0 only two imino signals belonging to homodimer persist, while third signal at δ 13.10 ppm is significantly reduced. Moreover, signal corresponding to hairpin at δ 12.41 ppm is absent. Lowering of pH to 4.0 results in almost complete disappearance of signals indicating guanine imino protons of homodimer and hairpin. Signals belonging to homodimer and hairpin reappear after increasing pH back to 6.0, showing reversibility.

Homodimer–hairpin equilibrium is preserved in concentration range between 0.05 and 1.1 mM of r(G_4_C_2_)_4_ at pH 6.0 and 25 °C based on integral ratios of imino signals in ^1^H-NMR spectra (Figure S15). Homodimer–hairpin equilibrium was confirmed by 20% native PAGE at pH 6.0, 5 °C and 0.3, 0.2 and 0.1 mM oligonucleotide concentrations per strand (Figure S16). Slower migrating band at 35 bp corresponds to homodimer, while constructs with band at 18 bp affirms hairpin formation.

### 2.3. Formation of r(G_4_C_2_)_4_ G-Quadruplex is Favoured by Annealing in the Presence of K^+^ Ions

^1^H-NMR spectra of r(G_4_C_2_)_4_ acquired at 25 °C with increasing concentration of K^+^ ions show that homodimer and hairpin persist even after exposure of sample to 100 mM concentration of K^+^ ions for more than 7 days (Figure 3d). Only after annealing of the sample in the presence of 100 mM concentration of K^+^ ions signals belonging to imino protons of G-C base pairs within homodimer and hairpin almost disappear. At the same time signals between δ 10.5 and 11.5 ppm corresponding to imino protons of guanines involved in G-quartets appear in ^1^H-NMR spectrum. D_t_ values of aromatic proton signals at 25 °C were in range between 1.1 and 1.5 × 10^−10^ m^2^s^−1^, which suggesting formation of G-quadruplexes of different sizes.

### 2.4. Antisense hybridizes with sense RNA oligonucleotide

**Figure 4:**
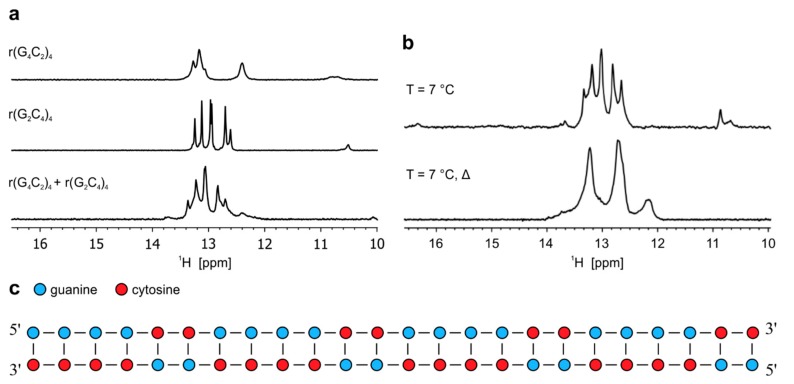
Formation of heterodimer by oligonucleotides r(G_4_C_2_)_4_ and r(G_2_C_4_)_4_. (**a**) ^1^H-NMR spectra of r(G_4_C_2_)_4_, r(G_2_C_4_)_4_ and their equimolar mixture (r(G_4_C_2_)_4_ + r(G_2_C_4_)_4_) at pH 6.0 and 25 °C. (**b**) ^1^H-NMR spectra (r(G_4_C_2_)_4_ + r(G_2_C_4_)_4_) at 7 °C before and after annealing of the sample. Δ represents NMR spectrum after annealing. All spectra were acquired at 0.2 mM oligonucleotide concentration per sense and antisense strand. **c**) Schematic presentation of heterodimer adopted by r(G_2_C_4_)_4_ and r(G_4_C_2_)_4_.

Imino region of the ^1^H-NMR spectrum of equimolar mixture of r(G_2_C_4_)_4_ and r(G_4_C_2_)_4_ recorded at pH 6.0 and 25 °C reveals five well resolved imino signals between δ 12.5 and 13.5 ppm, with slightly different chemical shifts as observed for imino signals of individual sense and antisense RNA corresponding to G-C base pairs of heterodimer (Figure 4a). In the same chemical shift range small overlapping imino signals of homodimers formed individually by r(G_2_C_4_)_4_ and r(G_4_C_2_)_4_ can still be observed, while the presence of small imino signal at δ 12.45 ppm corresponds to hairpin of r(G_4_C_2_)_4_. Decrease in temperature to 7 °C leads to observation of additional signals between δ 10.5 and 11.0 ppm corresponding to N1-carbonyl G-G base pairs, which are stabilized within homodimer of r(G_4_C_2_)_4_. Signal of low intensity at δ 16.45 ppm corresponds to hemi-protonated C^+^-C base pairs that are part of hairpin formed by r(G_2_C_4_)_4_ (Figure 4b). Existence of a low population of hairpins in equimolar mixture was also confirmed with CD spectrum at pH 6.0 and 25 °C, where a shoulder at wavelength of 285 nm can be observed in addition to a negative signal at 212 and positive one at 265 nm (Figure S17) [20]. After annealing, signals corresponding to G-G base pairs and hemi-protonated C^+^-C base pairs disappear completely in ^1^H-NMR spectra acquired at pH 6.0 and 7 °C as well as 25 °C (Figure 4b and Figure S18). In CD spectrum recorded at pH 6.0 and 25 ˚C after annealing signals at wavelengths of 212 and 265 nm persist, while shoulder at 285 nm is significantly reduced (Figure S17). 2D DOSY acquired on annealed sample at pH 6.0 and 25 °C afforded D_t_ of 1.05 ± 0.05 × 10^−10^ m^2^s^−1^, which is in accordance with hydrodynamic properties of dimeric structure and calculated D_t_ value by HydroPro program. Therefore, secondary structure of heterodimer formed by r(G_2_C_4_)_4_//r(G_4_C_2_)_4_ consists of 24 Watson-Crick G-C base pairs with indications that some G-C base pairs are more flexible than others, which helps in interpretation of a broad nature of signals in ^1^H-NMR spectrum (Figure 4c and Figure S18).

Comparison of equimolar mixture of r(G_2_C_4_)_4_ and r(G_4_C_2_)_4_ with samples of individual RNA oligonucleotides on 20% native PAGE at pH 6.0 and 5 °C reveals a slow migrating band at approximately 40 bp, which corresponds to heterodimer (Figure S19). Low-intensity bands corresponding to hairpins formed by individual RNA oligonucleotides are also present at approximately 18 and 15 bp.

**Figure 5:**
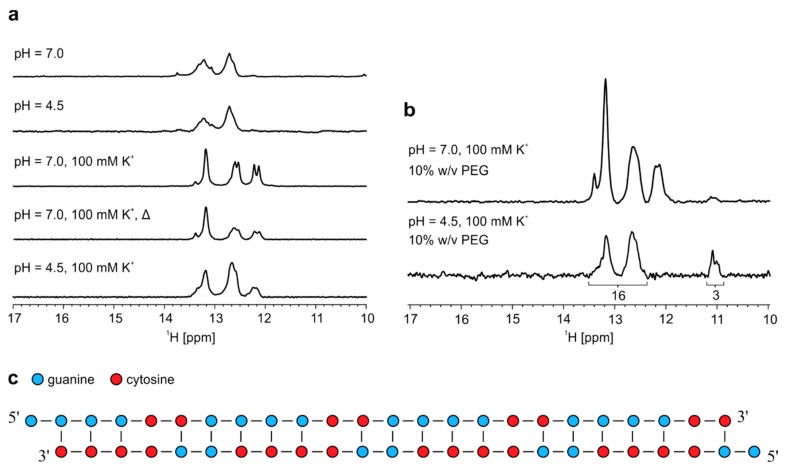
Effect of pH, presence of K^+^ ions and PEG on heterodimer. (**a**) ^1^H-NMR spectra of annealed equimolar mixture of r(G_4_C_2_)_4_ and r(G_2_C_4_)_4_. Δ represents NMR spectrum after second annealing. All spectra were acquired on an 800 MHz NMR spectrometer at 25 °C and 0.2 mM oligonucleotide concentration per strand. (**b**) ^1^H-NMR spectra of annealed equimolar mixture of r(G_4_C_2_)_4_ and r(G_2_C_4_)_4_ in the presence of 100 mM K^+^ and PEG at different pHs acquired at 25 °C and 0.2 mM oligonucleotide concentration per sense and antisense strand. Numbers under signals in the bottom spectrum represent integral values. **c**) Proposed model of heterodimer in the presence of 10% *w*/*v* PEG at pH 4.5.

Heterodimer formed by r(G_2_C_4_)_4_ and r(G_4_C_2_)_4_ was exposed to low pH and to the presence of K^+^ ions that could induce structural changes leading to formation of i-motifs and G-quadruplexes, respectively. However, lowering of pH did not result in any significant difference of imino signals in ^1^H-NMR spectrum (Figure 5a). On the other hand, addition of K^+^ ions to 100 mM concentration at pH 7.0 leads to additional stabilization of G-C base pairs since two new signals between δ 12.0 and 12.5 ppm appear. Imino signals that would indicate G-quadruplex formation are not observed even after additional annealing performed at 100 mM KCl (Figure 5a). Decrease in pH to 4.5 at 100 mM K^+^ ions leads to slightly less distinct imino signals with no apparent change in their chemical shifts. Addition of PEG at pH 7.0 and 100 mM K^+^ ions did not promote G-quadruplex formation as signals belonging to heterodimer between δ 12.0 and 13.5 ppm remain unchanged (Figure 5b). Two imino signals corresponding to G-C base pairs at δ 12.12 and 12.20 ppm disappear after lowering pH to 4.5 in the presence of PEG, while the intensity of signal at δ 13.40 ppm is significantly reduced. At the same time, signals most likely corresponding to N1-carbonyl G-G base pairs appear between δ 10.9 and 11.2 ppm, since signals corresponding to guanine imino protons within such base pairs are stabilized by lower pH [22]. Relative ratio of integrals of imino signals corresponding to G-C base pairs between δ 12.2 and 13.6 ppm and G-G base pairs between δ 10.9 and 11.2 ppm is 16:3. At low pH, it would be expected that signals corresponding to imino protons are more intense due to slower exchange with protons of bulk water in comparison to higher pH. Based on this ratio we concluded that heterodimer exposed to low pH and PEG most likely experienced structural changes, which resulted in one base pair shift, causing a reduction of initial 24 G-C base pairs to 16 G-C base pairs and increase from zero to three G-G base pairs (Figure 5c).

## 3. Discussion

Here we have shown that sense and antisense RNA oligonucleotides containing four hexanucleotide repeats of pathological repeat expansion of *C9orf72* gene can adopt diverse structures depending on the conditions of solution. ^1^H-NMR and CD spectra together with diffusion data at pH 7.0 and 25 °C indicate that r(G_2_C_4_)_4_ forms a homodimer with 16 Watson-Crick G-C base pairs. Homodimer was previously observed for closely related oligonucleotide r((C_4_G_2_)_3_C_4_) for which the crystal structure revealed 12 Watson-Crick G-C base pairs and 6 hemi-protonated C^+^-C base pairs [18]. Lowering of pH to 4.5 shifts the equilibrium towards formation of a hairpin with G-C base pairs, hemi-protonated C^+^-C base pairs and CCCG loop. This process was also observed in the presence of 10% *w*/*v* PEG, which was used to simulate molecular crowding conditions. The formation of hairpin adopted by r(C_4_G_2_)_4_ was previously proposed only through data obtained with CD spectra [17]. Although i-motif adopted by hexanucleotide repeats was previously described for antisense DNA oligonucleotides [1], it has never been observed for antisense RNA oligonucleotides comprising the repeat expansion(s). In the present study ^1^H-NMR spectra of r(G_2_C_4_)_4_ at pH 4.5 and 5 °C reveal the presence of imino signal at δ 15.75 ppm. According to its chemical shift value, this signal could correspond to imino protons of hemi-protonated C^+^-C base pairs within i-motif.

Previous studies of RNA dimers showed that G-G base pairs are the most stable compared to other mismatches [23]. However, single G-G base pair is more stable in comparison to two consecutive G-G base pairs likely due to an unfavorable stacking interaction between adjacent G-G pairs [24]. Our data show that r(G_4_C_2_)_4_ forms two structures, homodimer and hairpin, at pH 6.0 and 25 °C. Their equilibrium populations are temperature-dependent. Lowering the temperature favors the formation of homodimer and stabilization of its inherent G-G base pairs in N1 carbonyl symmetric geometry. Both structures are stable within narrow pH range between 5.0 and 6.0. While further decrease in pH leads to disappearance of all imino signals, increase to pH 7.0 favors homodimer. In contrast to cytosine-rich antisense oligonucleotide r(G_2_C_4_)_4_, guanine-rich sense oligonucleotide r(G_4_C_2_)_4_ has the potential to form secondary structures called G-quadruplexes. Several studies have shown that G-quadruplexes adopted by pathological RNA molecules influence many essential cell processes such as translation, splicing, nuclear transport and function of RNA-binding proteins [8,10,25,26,27]. In solution RNA G-quadruplex can be stabilized by various cations, which enable their distinct folding topologies [28]. To approach physiological conditions r(G_4_C_2_)_4_ was observed in the presence of K^+^ ions, which are the most abundant in cells. Even in the presence of 100 mM K^+^ ions for more than one week, r(G_4_C_2_)_4_ does not form G-quadruplexes. Only after annealing, during which homodimer and hairpin are almost completely unfolded, a group of new signals corresponding to imino protons of guanines involved in G-quartets appears at 25 °C, thus indicating the presence of G-quadruplexes. Latter observations are in agreement with recent spectroscopic data combined with chemical and enzymatic probing of r(G_4_C_2_) repeat expansion, which together suggest temperature-dependent equilibrium between stable hairpins formed at 37 °C and G-quadruplexes adopted at higher annealing temperatures [10,29]. Diffusion data also indicate that intra- and inter-molecular G-quadruplexes are present, which is in agreement with electrophoretic mobility shift assay where both G-quadruplexes adopted by r(G_4_C_2_)_4_ in the presence of 200 mM K^+^ ions at pH 7.4 were observed previously [17]. Additionally, ^1^H-NMR and CD spectroscopy together with results from UV melting experiments imply that oligonucleotide r((G_4_C_2_)_3_GGGGC) in the presence of 40 mM KCl forms a unimolecular four-stacked parallel-stranded G-quadruplex with propeller loops [16]. Furthermore, CD spectroscopy and RNase protection assay showed that r(G_4_C_2_GGGG) at 100 mM K^+^ ions adopt a dimeric three-stacked parallel-stranded G-quadruplex with propeller loops [10].

Transcription of the *C9orf72* gene occurs on both DNA strands leading to production of sense and antisense RNA transcripts, which are simultaneously present in the nucleus. Moreover, RNA foci of sense and antisense RNA transcripts appear to colocalize meaning that the presence of complementary strand could influence the structures formed independently by individual RNA transcripts [30,31]. In the present study ^1^H-NMR and CD spectra as well as PAGE indicate that sense and antisense RNA oligonucleotides form a heterodimer, while structures adopted by individual RNA oligonucleotide are still present as minor species. Exposure of heterodimer to physiological concentration of K^+^ ions as well as its annealing did not lead to G-quadruplex formation. In both cases, additional G-C base pairs within the heterodimer were stabilized, which implies that heterodimer is the favorable structure under these conditions. When equimolar mixture of DNA oligonucleotides d(G_2_C_4_)_8_ and d(G_4_C_2_)_8_ in solution with 100 mM K^+^ and low pH was exposed to PEG, signals of imino protons involved in G-quartets, G-C base pairs and hemi-protonated C^+^-C base pairs within i-motifs were observed [1]. On the contrary, RNA heterodimer formed by r(G_2_C_4_)_4_ and r(G_4_C_2_)_4_ does not undergo structural changes at pH 4.5 and formation of hemi-protonated C^+^-C base pairs associated with i-motif. Even in the presence of 100 mM K^+^ ions at pH 4.5, the heterodimer maintained its structure. The effect of low pH and K^+^ ions on heterodimer was also observed in the presence of PEG. While 100 mM K^+^ ion concentration in the presence of 10% *w*/*v* PEG at pH 7.0 did not promote G-quadruplex formation, the reduction of pH to 4.5 under these conditions led to destabilization of certain G-C base pairs and most likely stabilization of N1-carbonyl G-G base pairs resulting in formation of heterodimer with one base pair shift.

Finally, described RNA structures and dynamic equilibria they are involved in could represent potential drug targets for modulation of DPRs and RNA toxicity-dependent mechanism. Structures of sense and antisense RNA transcripts could play a key role in translation where the production of DPRs could be initiated or repressed through formation of protein-binding hairpins, i-motifs and G-quadruplexes [10]. Recently, small binding molecules have been identified that reduce the overall RNA foci and DPRs by specifically targeting G_4_C_2_ repeat RNA G-quadruplexes, which ultimately prolonged the life expectancy of ALS/FTLD fruit fly model [27]. In addition, study on interaction between G-quadruplex adopted by r(G_4_C_2_)_8_ and porphyrin TMPyP4 has shown that TMPyP4 distorts the formation of G-quadruplex, which prevents binding of proteins associated with ALS such as hnRNPA1 [32]. Self-dimerization, intra- and inter-molecular association of described structures could, therefore, play an important part in formation of RNA foci and sequestration of RNA-binding proteins. Over the years, several studies targeting guanine-rich sense RNA transcripts with antisense DNA oligonucleotides (ASOs) turned out to be more or less successful [33,34]. ASOs targeting intronic region downstream of sense RNA repeats were developed, which reduced the overall RNA foci in fibroblasts and iPSC-derived neurons [8]. Furthermore, ASOs have been identified that significantly reduce pathogenic sense RNA foci but do not affect the level of C9orf72 encoding RNAs [35,36] In one case reduction of RNA foci using ASO816 also led to significant correction of abnormally expressed genes after treatment of ALS patient iPSC-derived motor neurons [36]. However, designing an ASO that would specifically target and reduce antisense RNA foci remains a challenge for future studies. On the other hand, RNA duplexes were constructed, which were able to target and degrade both sense and antisense RNA foci by exploiting the RNAi pathway [37]. Although ASOs and RNAi show immense promise in treatment of ALS and FTLD, half-life of RNA duplexes and long term effects of ASOs needs to be carefully assessed. Therefore, the complexity of pathological RNA molecules of *C9orf72* repeat expansion associated with ALS and FTLD demands further comprehensive studies as structures adopted by RNA transcripts act not only as important promoters of RNA toxicity-dependent mechanism but also present promising therapeutic targets in ALS, FTLD and other repeat-associated neurodegenerative diseases.

## 4. Materials and Methods

### 4.1. Sample Preparation

Reverse-phase HPLC purified RNA oligonucleotides r(G_2_C_4_)_4_ and r(G_4_C_2_)_4_ were purchased from Eurogentec (Seraing, Belgium). After addition of 5 mL of 2 M LiCl samples were heated to 90 °C for 5 min. Samples were extensively dialyzed against autoclaved H_2_O through four 30 min cycles and then concentrated using an Amicon ultrafilter (Merck Millipore, Herfordshire, UK). Following the overnight lyophilization, samples were diluted in 90% of autoclaved H_2_O and 10% of ^2^H_2_O. The concentrations of the 300 μL NMR samples were 0.9, 0.7 and 0.3 mM for r(G_2_C_4_)_4_ and 1.1, 0.7, 0.3, 0.2, 0.1 and 0.05 mM for r(G_4_C_2_)_4_. In the case of equimolar mixing experiments, equimolar quantities of r(G_2_C_4_)_4_ and r(G_4_C_2_)_4_ were diluted to final oligonucleotide concentration of 0.2 mM per sense and antisense strand. Samples of r(G_4_C_2_)_4_ and mixed RNA oligonucleotides were also heated to 90 °C for 5 min prior to annealing. For molecular crowding experiments, sample of r(G_2_C_4_)_4_ and mixed RNA oligonucleotides were diluted in 10% *w*/*v* PEG (8000 MW) (Sigma Aldrich, Munich, Germany) in autoclaved H_2_O to 0.3 and 0.2 mM oligonucleotide concentration per strand, respectively. The pH of samples was adjusted by the addition of 0.1 mM LiOH or HCl.

### 4.2. Circular Dichroism Spectroscopy

CD spectra were acquired on Applied Photophysics Chirascan CD spectrometer using a 0.1 cm path length quartz cell. The wavelength was varied from 200 to 320 nm with 0.5 nm per step. Time-per-point was set to 0.5 s. A blank containing only autoclaved H_2_O was used for baseline correction. For CD spectroscopy the sample of r(G_2_C_4_)_4_ was measured immediately after dilution in autoclaved H_2_O at 5, 25, and 37 °C, at pH 7.0. Measurements at different pHs were performed in autoclaved H_2_O at 25 °C and pH 4.5, 5.0, 5.5, 6.0, 6.5 and 7.0. The pH of samples was adjusted by the addition of 0.1 mM LiOH or HCl. CD spectra of equimolar mixture of r(G_2_C_4_)_4_ and r(G_4_C_2_)_4_ in autoclaved H_2_O before and after annealing were recorded at 25 °C. CD measurements were carried out on a sample of r(G_2_C_4_)_4_ and mixed RNA oligonucleotides diluted to concentration of 100 μM per sense and antisense strand.

### 4.3. UV Spectroscopy

Melting experiments of r(G_2_C_4_)_4_ and r(G_4_C_2_)_4_ were performed on a Varian Cary 100 Bio UV-VIS spectrometer (Varian Inc.) equipped with a thermoelectric temperature controller. UV melting experiments were performed on samples diluted to 2.6, 3.0, 7.7, 9.3 and 20 μM oligonucleotide concentration per strand in 20 mM lithium cacodylate buffer at pH 5.0 and 7.0. Each sample was placed in 1, 0.5 or 0.1 cm path-length quartz cell. Mineral oil and a tightly fixed cuvette cap were used to seal the quartz cells in order to prevent evaporation and sample loss due to high temperatures. To prevent condensation at lower temperatures a stream of nitrogen was applied throughout the measurements. Folding/unfolding processes were followed between 20 and 90 °C by measuring absorbance at 260 nm. Data were acquired using scan rate of 0.1 °C min^−1^. Temperature of half transition (T_1/2_) for homodimer was determined using first derivative method, while temperature of half transition for hairpin was estimated from obtained melting curves.

### 4.4. Native PAGE

Native PAGE of r(G_2_C_4_)_4_, r(G_4_C_2_)_4_ and equimolar mixture of r(G_2_C_4_)_4_ and r(G_4_C_2_)_4_ was performed on 20% polyacrylamide gel (5 °C at 100 V) in 1×TBE (pH 6.0) buffer. Samples for native PAGE were prepared by mixing 5 or 10 μL of stock or diluted RNA oligonucleotide solution, 5 μL of loading buffer 15% Ficoll and diluted to 20 μL with autoclaved H_2_O. The approximate band size was determined by using the GeneRuler Ultra Low range DNA Ladder (Thermo Scientific, Waltham, MA, USA). The samples were treated at room temperature overnight before loading, except for the equimolar mixture sample, which was heated to 90 °C and cooled slowly at room temperature prior to loading. Following the overnight electrophoresis, the gel was stained with Stains-All gel stain solution (Sigma Aldrich).

### 4.5. NMR Spectroscopy

NMR spectra were obtained with Agilent Technologies VNMRS 800 MHz and DD2 600 MHz NMR spectrometers equipped with triple resonance cold probe. Standard 1D ^1^H-NMR spectra were acquired with the use of DPFGSE or watergate 3919 solvent suppression. Diffusion coefficient measurements were performed by a spin-echo pulse sequence with PFG gradient strengths between 1.3 and 32.5 Gcm^−1^. NOESY spectra were recorded using mixing times of 100, 80, 40 and 20 ms. TOCSY spectrum was recorded using mixing times of 80 ms. ^1^H-NMR spectra were acquired at 0, 5, 7, 15, 20, 25, 37 and 40 °C. Effect of different pHs was observed by recording ^1^H-NMR spectra at pH 4.5, 5.0, 5.5, 6.0, 6.5 and 7.0. Effect of molecular crowding conditions was observed by recording ^1^H-NMR spectra in the presence of 10% *w*/*v* PEG (8000 MW) in autoclaved H_2_O. In experiments with K^+^ ions 3M KCl was diluted into the sample up to the desired concentration of K^+^ ions. 1D and 2D NMR spectra were processed and analysed using VNMRJ (Varian Inc.), MestReNova and Sparky (UCSF) software. All NMR spectra were acquired on 600 MHz NMR spectrometer unless stated otherwise. DSS (4,4-dimethyl-4-silapentane-1-sulfonic acid) was used as external reference in all NMR spectra.

## 5. Conclusions

Exploiting NMR and complementary methods allowed determination of structures adopted by guanine rich r(G_4_C_2_)_4_ sense and cytosine rich r(G_2_C_4_)_4_ antisense RNA oligonucleotides associated with ALS and FTLD. Under conditions approaching physiological relevance, both sense and antisense RNA oligonucleotides form dimers and hairpins. Equilibrium of structures adopted by antisense RNA oligonucleotides is pH-dependent, hence lowering the pH favours hairpin formation. On the other hand, equilibrium of structures adopted by sense RNA oligonucleotides is temperature-dependent owing to prevalent formation of dimer at lower temperatures. Furthermore, sense RNA oligonucleotides form structurally different G-quadruplexes in the presence of KCl and antisense RNA oligonucleotides indicate i-motif formation at low pH and temperature. Simultaneous presence of both RNA oligonucleotides revealed formation of heterodimers although structures adopted by individual RNA oligonucleotides seem to persist in the minority. Adopted structures of pathological RNA oligonucleotides could play a key role in sequestering RNA binding proteins and, therefore, represent an important biological target of drug design in the combat against ALS and FTLD.

## Supplementary Materials

The following are available online, Figure S1: Canonical and non-canonical base pairs, Figure S2: Cytosine H5/H6 correlation region of 2D TOCSY NMR spectrum of r(G_2_C_4_)_4_, Figure S3: ^1^H-NMR spectra of r(G_2_C_4_)_4_ in 10% ^2^H_2_O recorded at 5, 25 and 37 ˚C, Figure S4: UV melting experiment of homodimer adopted by r(G_2_C_4_)_4_, Figure S5: Imino regions of ^1^H-NMR spectra of r(G_2_C_4_)_4_ in 10% ^2^H_2_O at different pH, Figure S6: Imino regions of ^1^H-NMR spectra of r(G_2_C_4_)_4_ in 10% ^2^H_2_O at different pH and in the presence of 10% w/v PEG, Figure S7: CD spectra of r(G_2_C_4_)_4_ with pH ranging from 4.5 to 7.0, Figure S8: 20% native PAGE of r(G_2_C_4_)_4_ at pH 6.0 and 5 ˚C, Figure S9: Imino-imino spectral region of 2D NOESY spectrum of r(G_2_C_4_)_4_, Figure S10: Imino regions of ^1^H-NMR spectra of r(G_2_C_4_)_4_ in 10% ^2^H_2_O at 5, 25 and 40 ˚C, Figure S11: UV melting experiment of hairpin adopted by r(G_2_C_4_)_4_, Figure S12: ^1^H-NMR spectra of r(G_4_C_2_)_4_ in 10% ^2^H_2_O at 0, 7, 15, 20, 25 and 37 ˚C, Figure S13: Imino regions of ^1^H-NMR spectra of r(G_4_C_2_)_4_ in 10% ^2^H_2_O at 0 and 25 ˚C, Figure S14: Imino and aromatic regions of ^1^H-NMR spectra of r(G_4_C_2_)_4_ in 10% ^2^H_2_O at different pH, Figure S15: Imino regions of ^1^H-NMR spectra of r(G_4_C_2_)_4_ in 10% ^2^H_2_O at concentrations of 0.05, 0.1, 0.2, 0.3, and 1.1 mM per strand, Figure S16: 20% native PAGE of r(G_4_C_2_)_4_ at pH 6.0 and 5 ˚C, Figure S17: CD spectra of equimolar mixture of r(G_2_C_4_)_4_ and r(G_4_C_2_)_4_ before and after annealing, Figure S18: ^1^H-NMR spectra of equimolar mixture of r(G_2_C_4_)_4_ and r(G_2_C_4_)_4_ before and after annealing, Figure S19: 20% native PAGE of r(G_4_C_2_)_4_, r(G_2_C_4_)_4_ and r(G_4_C_2_)_4_ + r(G_2_C_4_)_4_ at pH 6.0 and 5 ˚C, Table S1: Translational diffusion coefficients (D_t_) of r(G_2_C_4_)_4_ at different pH values.
