# Supplementary material for: Structural Diversity of Sense and Antisense RNA Hexanucleotide Repeats Associated with ALS and FTLD
Source: Molecules. 2020 Jan 25;25(3):525. doi: 10.3390/molecules25030525 (PMC7037139; doi:10.3390/molecules25030525)
Supplement: Supplementary file 1 [file molecules-25-00525-s001.pdf]

molecules

Supplementary Materials

Structural diversity of sense and antisense RNA hexanucleotide repeats associated with ALS and FTLD

Tim Božič [1], Matja Zalar [1], Boris Rogelj [2,3], Janez Plavec [1,4,5,*] and Primož Šket [1,*]

[1] Slovenian NMR Centre, National Institute of Chemistry, Hajdrihova 19, SI-1000 Ljubljana, Slovenia.
[2] Department of Biotechnology, Jožef Stefan Institute, Jamova 39, SI-1000 Ljubljana, Slovenia.
[3] Biomedical Research Institute BRIS, Puhova 10, SI-1000 Ljubljana, Slovenia.
[4] EN-FIST Center of Excellence, Trg OF 13, SI-1000 Ljubljana, Slovenia.
[5] Faculty of Chemistry and Chemical Technology, University of Ljubljana, Večna pot 113, SI-1000 Ljubljana, Slovenia.
* Correspondence and requests for materials should be addressed to P. Šket (email: primoz.sket@ki.si) and J. Plavec (email: janez.plavec@ki.si)

Table of contents

**Figure S1**.	Canonical and non-canonical base pairs.	S2
**Figure S2**.	Cytosine H5/H6 correlation region of 2D TOCSY NMR spectrum of r(G_2C_4)$_4$.	S3
**Figure S3.**	^{1}H NMR spectra of r(G_2C_4)$_4$ in 10% ^2H_2O recorded at 5, 25 and 37 °C.	S3
**Figure S4.**	UV melting experiment of homodimer adopted by r(G_2C_4)$_4$.	S4
**Figure S5.**	Imino region of ^{1}H NMR spectra of r(G_2C_4)$_4$ in 10% ^2H_2O at different pH.	S5
**Table S1.**	Translational diffusion coefficients (D_t) of r(G_2C_4)$_4$ at different pH values.	S5
**Figure S6**.	Imino region of ^{1}H NMR spectra of r(G_2C_4)$_4$ in 10% ^2H_2O at different pH and in the presence of 10% w/v PEG.	S6
**Figure S7**.	CD spectra of r(G_2C_4)$_4$ with pH ranging from 4.5 to 7.0.	S6
**Figure S8.**	20% native PAGE of r(G_2C_4)$_4$ at pH 6.0 and 5 °C.	S7
**Figure S9.**	Imino-imino spectral region of 2D NOESY spectrum of r(G_2C_4)$_4$.	S7
**Figure S10.**	Imino regions of ^{1}H NMR spectra of r(G_2C_4)$_4$ in 10% ^2H_2O at 5, 25 and 40 °C.	S8
**Figure S11.**	UV melting experiment of hairpin adopted by r(G_2C_4)$_4$.	S9
**Figure S12.**	^{1}H NMR spectra of r(G_4C_2)$_4$ in 10% ^2H_2O at 0, 7, 15, 20, 25 and 37 °C.	S10
**Figure S13.**	Imino regions of ^{1}H NMR spectra of r(G_4C_2)$_4$ in 10% ^2H_2O at 0 and 25 °C.	S10
**Figure S14.**	Imino and aromatic region of ^{1}H NMR spectra of r(G_4C_2)$_4$ in 10% ^2H_2O at different pH.	S11
**Figure S15.**	Imino regions of ^{1}H NMR spectra of r(G_4C_2)$_4$ in 10% ^2H_2O at concentration of 0.05, 0.1, 0.2, 0.3, and 1.1 mM per strand.	S12
**Figure S16.**	20% native PAGE of r(G_4C_2)$_4$ at pH 6.0 and 5 ˚C.	S12
**Figure S17.**	CD spectra of equimolar mixture of r(G_2C_4)$_4$ and r(G_4C_2)$_4$ before (black) and after (red) annealing.	S13
**Figure S18.**	^{1}H NMR spectra of equimolar mixture of r(G_2C_4)$_4$ and r(G_2C_4)$_4$ before and after annealing.	S13
**Figure S19.**	20% native PAGE of r(G_4C_2)$_4$, r(G_2C_4)$_4$ and r(G_4C_2)$_4$ + r(G_2C_4)$_4$ at pH 6.0 and 5 °C.	S13

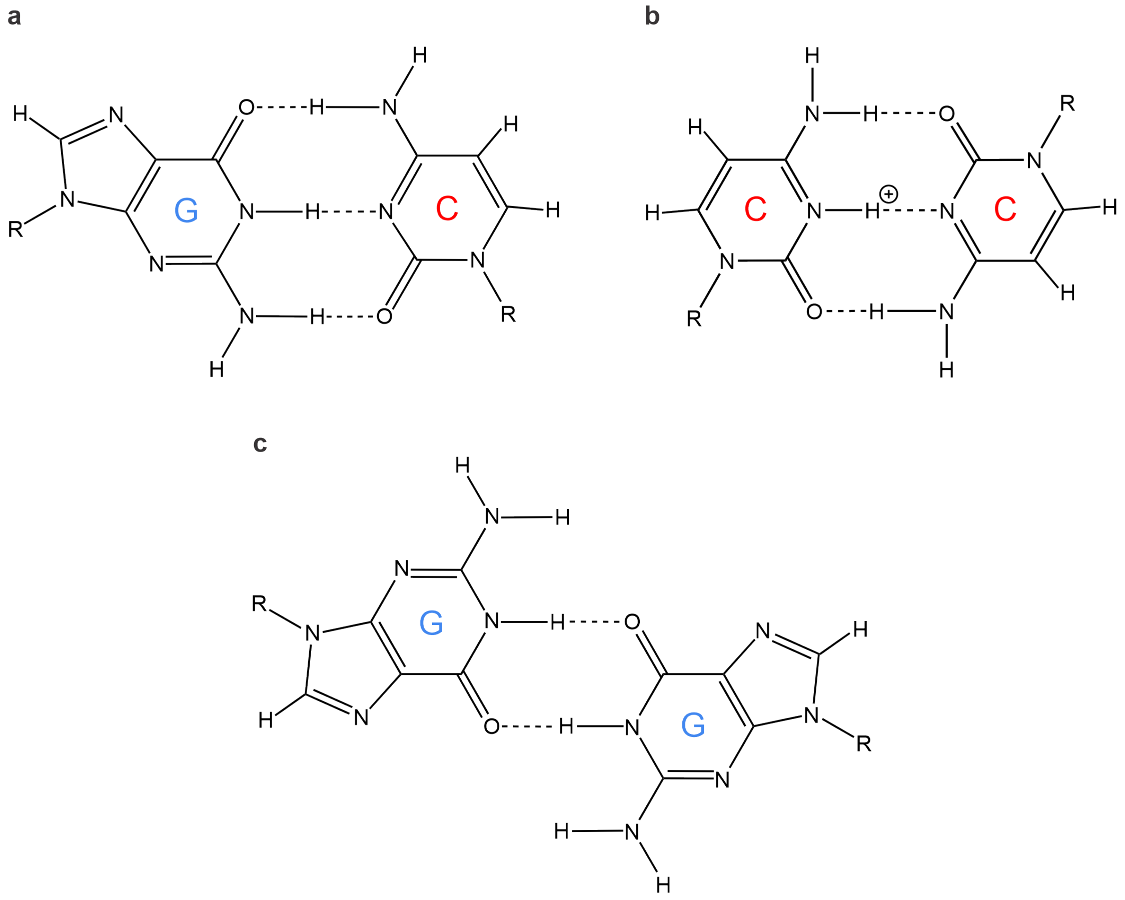

Figure S1. Canonical and non-canonical base pairs. Schematic presentation of **a**) Watson-Crick G-C base pair, **b**) hemi-protonated C^{+}-C base pair and **c**) G-G base pair in N1-carbonyl symmetric geometry.

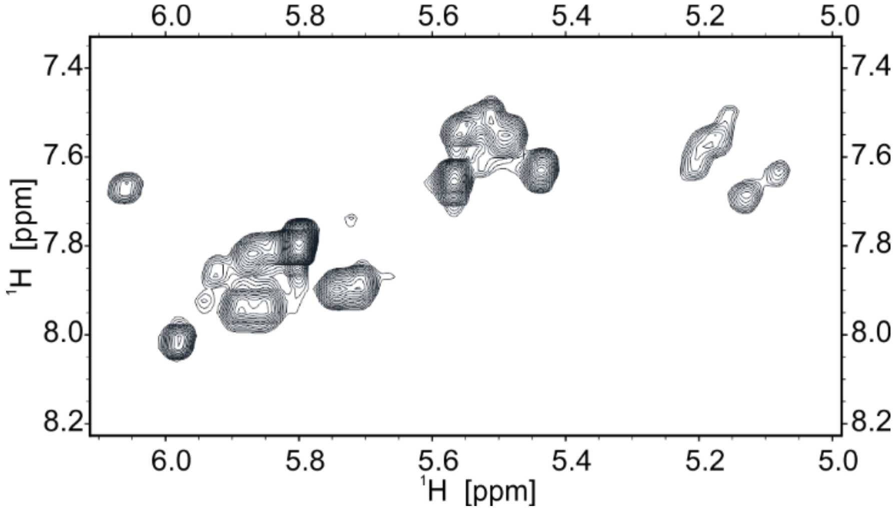

Figure S2. Cytosine H5/H6 correlation region of 2D TOCSY NMR spectrum of r(G_2C_4)$_4$. Spectrum was recorded at pH 7.0 and 25 °C at mixing time of 80 ms and concentration of 0.9 mM per strand.

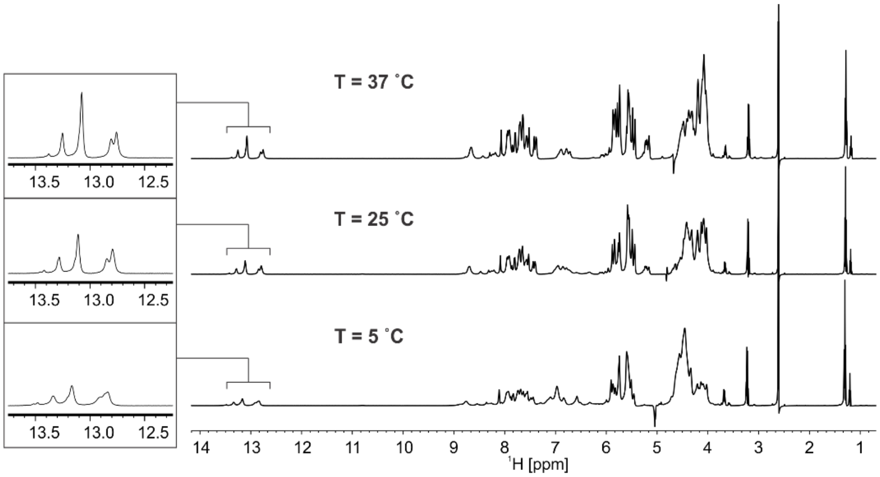

Figure S3. ^{1}H NMR spectra of r(G_2C_4)$_4$ in 10% ^2H_2O recorded at 5, 25 and 37 °C. At 25 ˚C the pH was 7.0. All NMR spectra were acquired at concentration of 0.9 mM per strand.

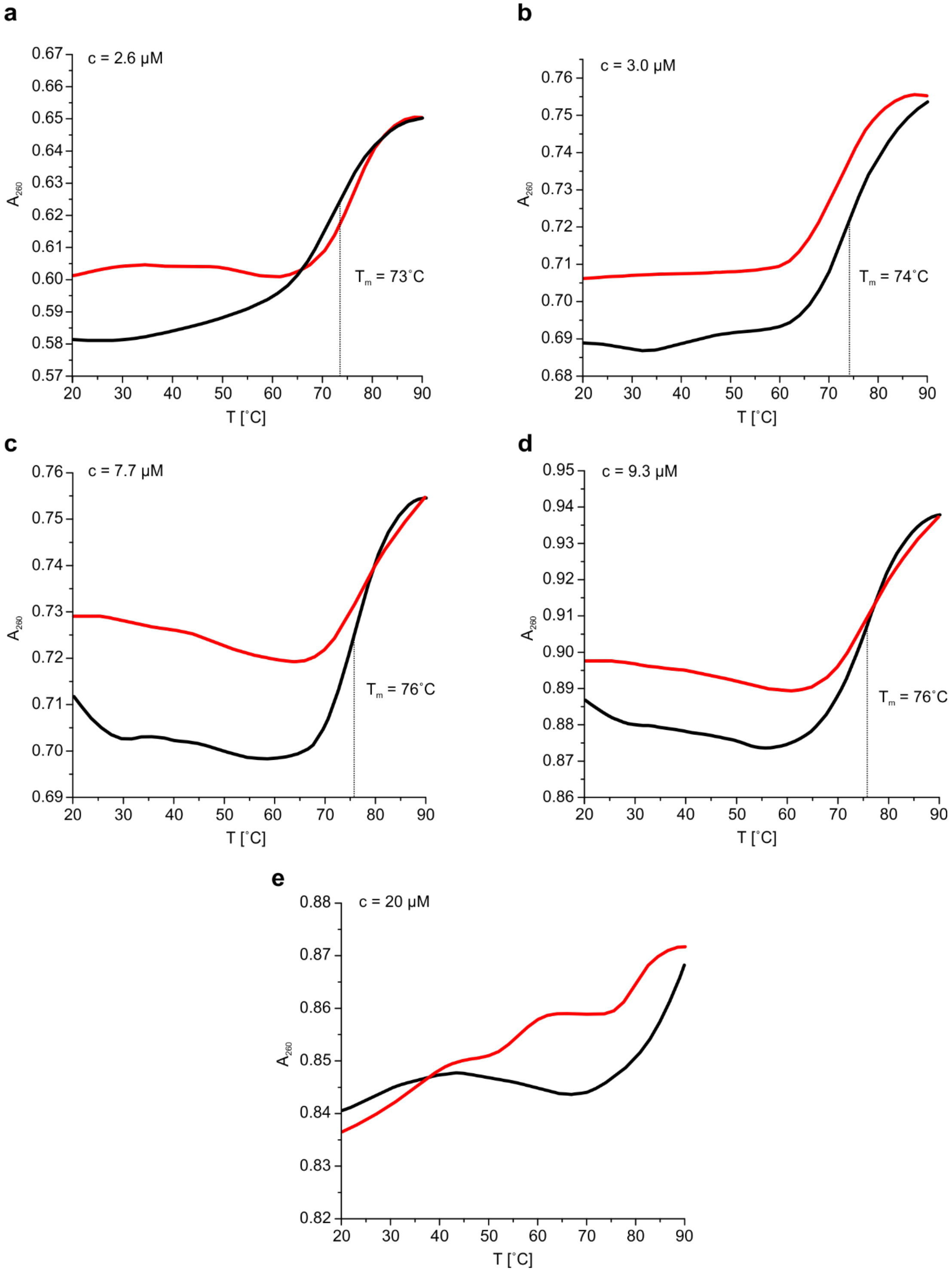

Figure S4. UV melting experiment of homodimer adopted by $r(G_2C_4)_4$. The absorbance at 260 nm was measured during one cycle of heating (black) and cooling (red) the sample in the temperature range between 20 and 90 °C. Temperature of half transition for homodimer adopted by $r(G_2C_4)_4$ was determined for all four oligonucleotide concentrations from melting curve obtained during heating. All samples were prepared in 20 mM Li^+ cacodylate buffer with pH 7.0. Concentrations of $r(G_2C_4)_4$ were **a**) 2.6, **b**) 3.0, **c**) 7.7, **d**) 9.3 and **e**) 20 μM per strand.

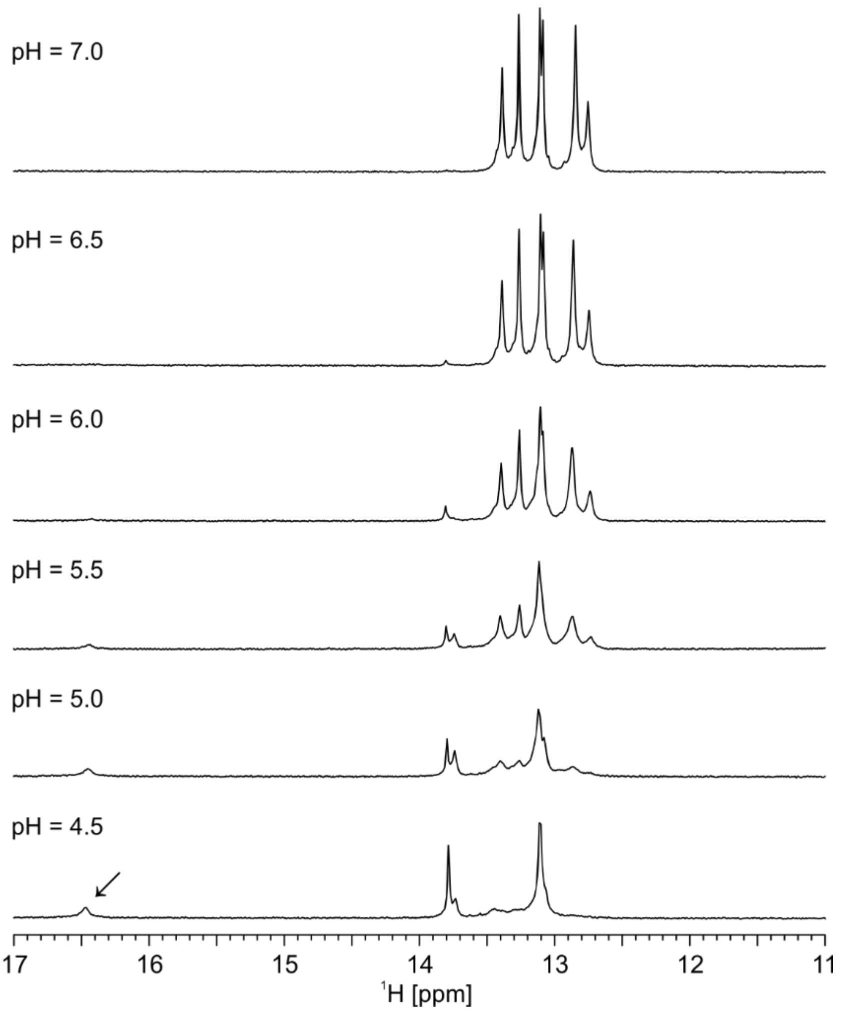

Figure S5. Imino region of ^{1}H NMR spectra of $r(G_2C_4)_4$ in 10% $^{2}H_2O$ at different pH. All NMR spectra were acquired at 25 °C and 0.3 mM oligonucleotide concentration per strand. Signal corresponding to hemi-protonated C^{+}-C base pairs is marked with an arrow.

Table 1. Translational diffusion coefficients (D_t) of $r(G_2C_4)_4$ at different pH values. All 2D DOSY spectra were acquired at 25 °C and concentration of 0.3 mM per strand. Standard deviation of the data set was ± 0.05.

pH	D_t [* 10^(−10) m^2s^{-1}]
7.0	0.91
6.5	0.94
6.0	1.25
5.5	1.30
5.0	1.35
4.5	1.40

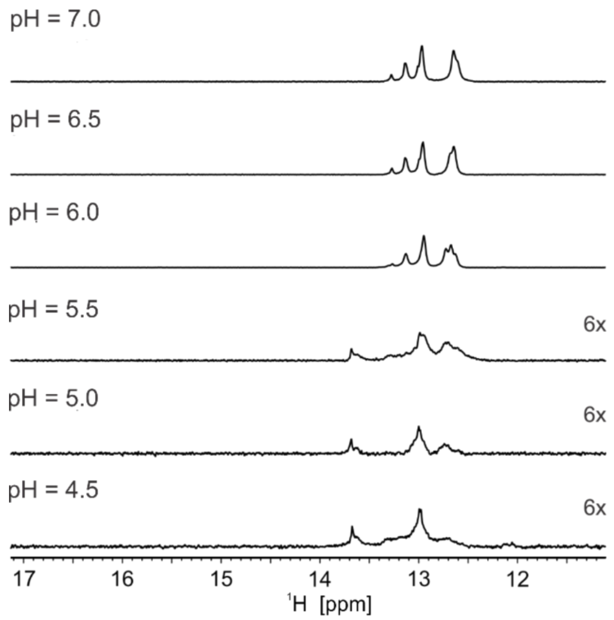

Figure S6. Imino region of ¹H NMR spectra of r(G_2C_4)$_4$ in 10% ^2H_2O at different pH and in the presence of 10% *w*/*v* PEG. All NMR spectra were acquired at 25 °C and concentration of 0.3 mM per strand. The vertical scales of the spectra with pH ranging from 5.5 to 4.5 have been increased six-fold (6×).

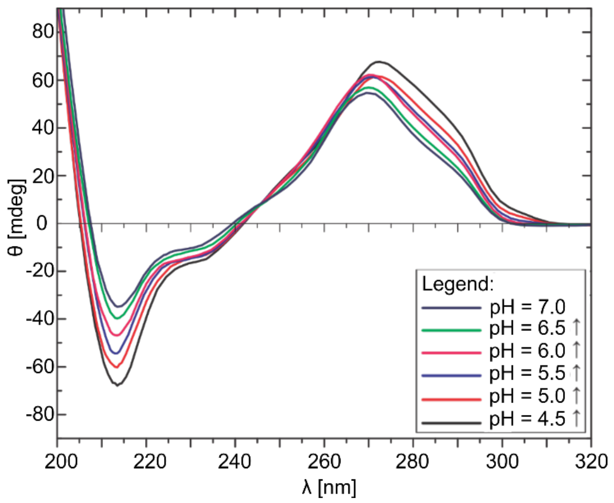

Figure S7. CD spectra of r(G_2C_4)$_4$ with pH ranging from 4.5 to 7.0. Arrows represent the direction of change in pH. All CD spectra were acquired at 25 °C and concentration of 100 μM per strand.

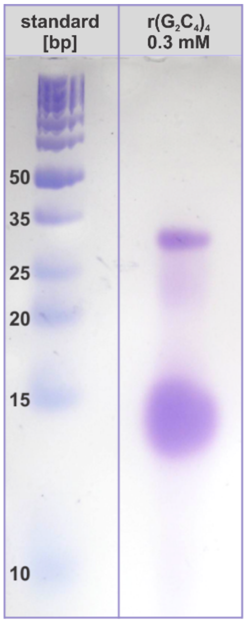

Figure S8. 20% native PAGE of $r(G_2C_4)_4$ at pH 6.0 and 5 °C. Concentration of $r(G_2C_4)_4$ was 0.3 mM per strand. DNA ladder was used as a standard.

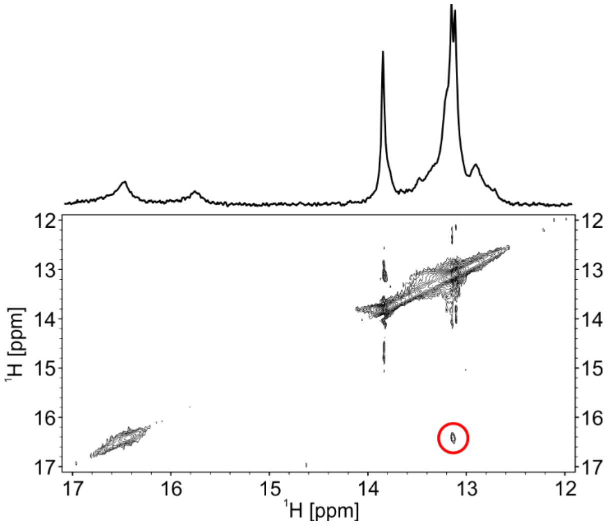

Figure S9. Imino-imino spectral region of 2D NOESY spectrum of $r(G_2C_4)_4$. Spectrum was recorded at pH 4.5 and 5 °C at mixing time of 100 ms and concentration of 0.7 mM per strand. Above 2D NOESY spectrum is 1D ^{1}H NMR spectrum of $r(G_2C_4)_4$ recorded at pH 4.5, 5 °C and oligonucleotide concentration of 0.7 mM per strand. NOE contact (marked with red circle) confirms the presence of hemi-protonated C^+-C base pairs within the hairpin.

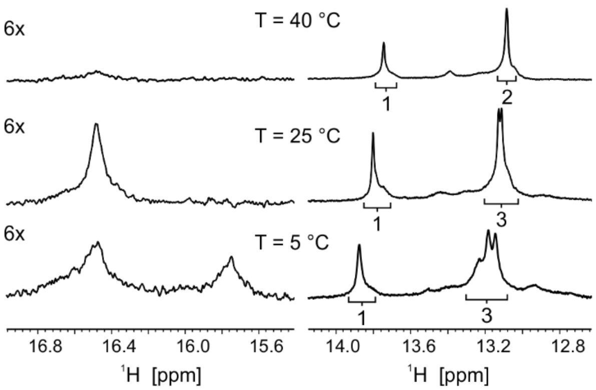

Figure S10. Imino regions of ^1H NMR spectra of r(G_2C_4)$_4$ in 10% ^2H_2O at 5, 25 and 40 °C. All NMR spectra were acquired at concentration of 0.3 mM per strand. The pH was 4.5 at 25 °C. Numbers under signals represent integral values. The vertical scales of the spectral regions between δ 15.6 to 16.8 ppm have been increased six-fold (6×).

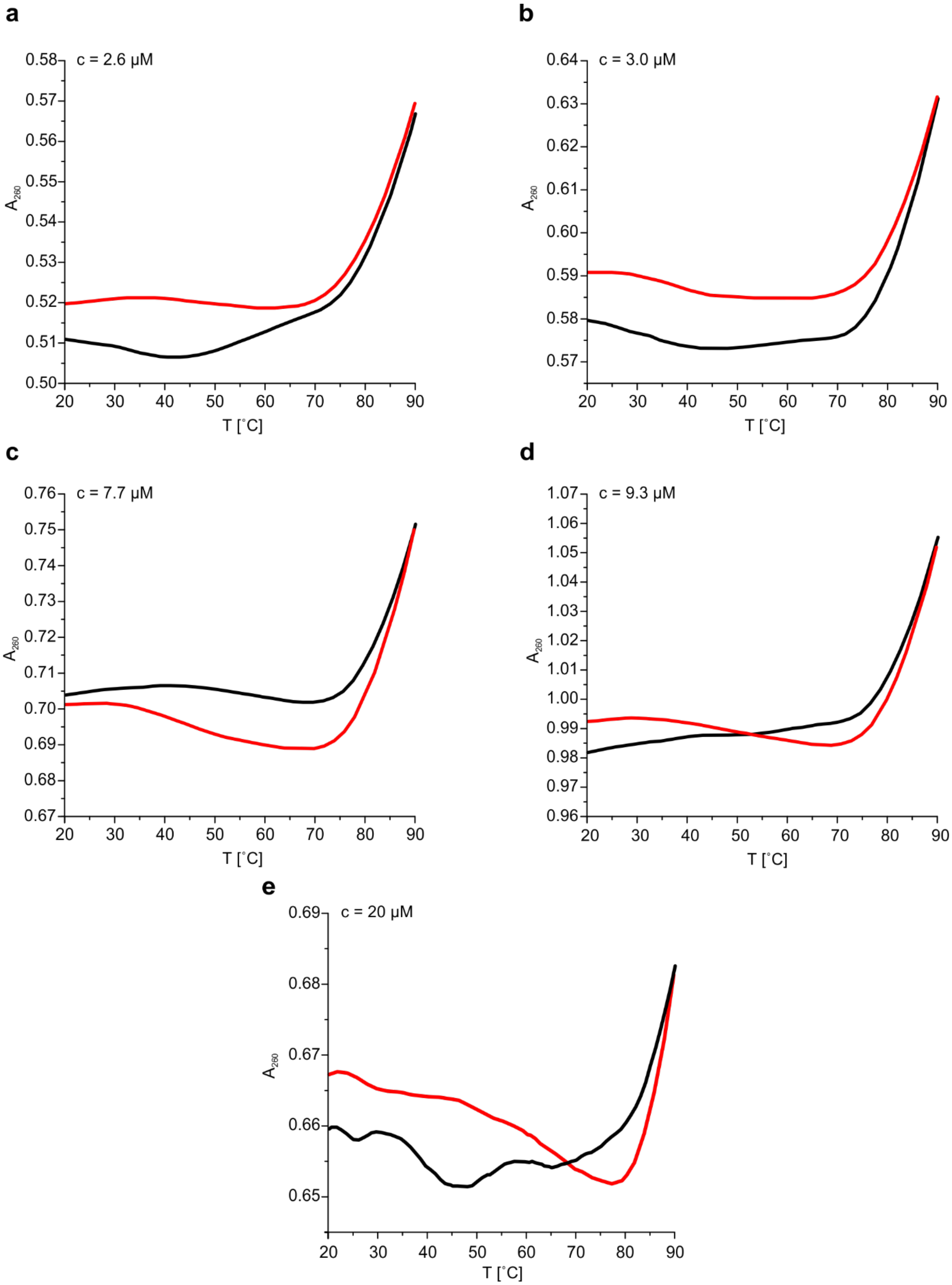

Figure S11. UV melting experiment of hairpin adopted by r(G_2C_4)$_4$. The absorbance at 260 nm was measured during one cycle of heating (black) and cooling (red) the sample in the temperature range between 20 and 90 °C. All samples were prepared in 20 mM Li^+ cacodylate buffer with pH 5.0. Concentrations of r(G_2C_4)$_4$ were **a**) 2.6, **b**) 3.0, **c**) 7.7, **d**) 9.3 and **e**) 20 μM per strand.

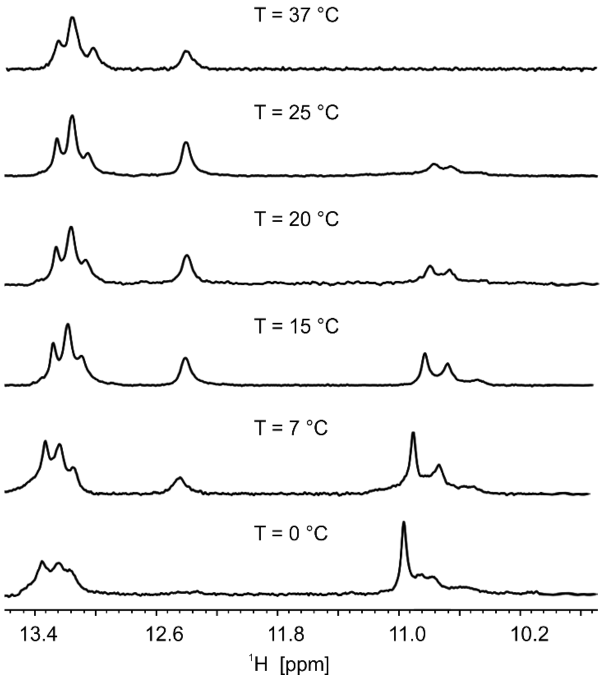

Figure S12. ^{1}H NMR spectra of r(G_4C_2)$_4$ in 10% ^2H_2O at 0, 7, 15, 20, 25 and 37 °C. All NMR spectra were acquired at concentration of 0.3 mM per strand. The pH was 6.0 at 25 °C.

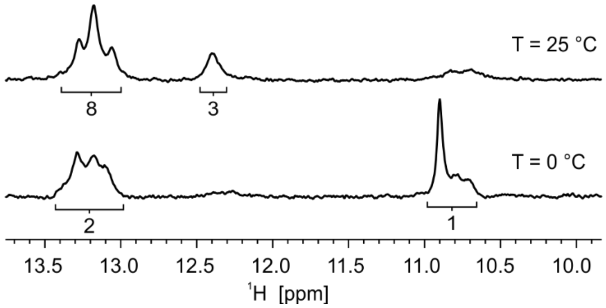

Figure S13. Imino regions of ^{1}H NMR spectra of r(G_4C_2)$_4$ in 10% ^2H_2O at 0 and 25 °C. Both NMR spectra were acquired at pH 6.0 and concentration of 0.3 mM per strand. Numbers under signals represent integral values.

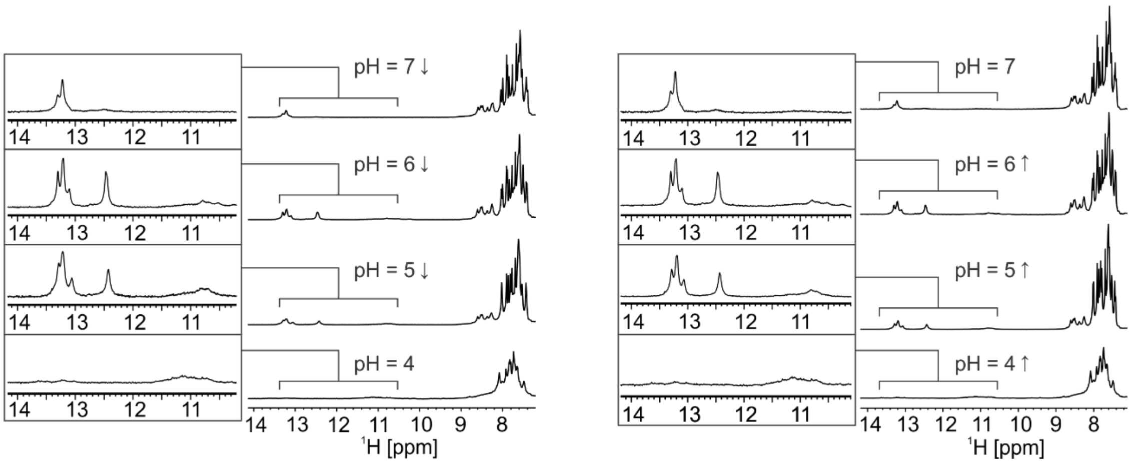

Figure S14. Imino and aromatic region of ^{1}H NMR spectra of r(G_4C_2)$_4$ in 10% ^2H_2O at different pH. Arrows represent the direction of change in pH. All NMR spectra were acquired on 800 MHz spectrometer at 25 °C and concentration of 0.3 mM per strand.

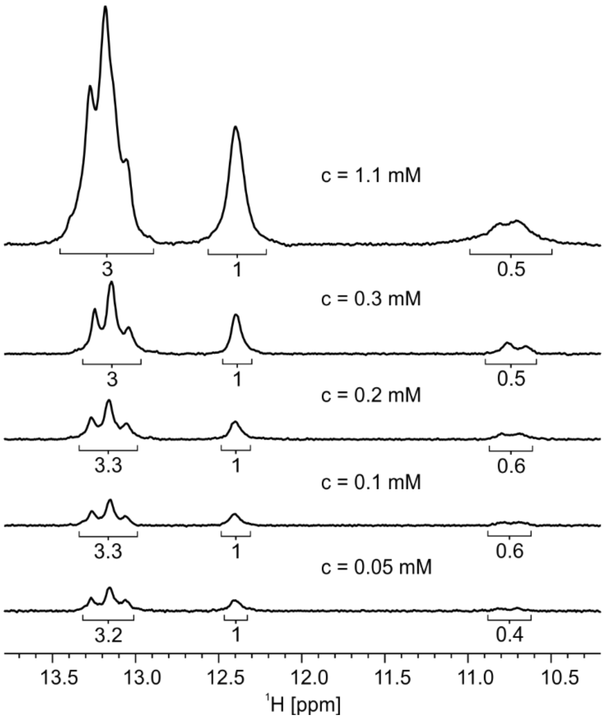

Figure S15. Imino regions of ^{1}H NMR spectra of r(G_4C_2)$_4$ in 10% ^2H_2O at concentration of 0.05, 0.1, 0.2, 0.3, and 1.1 mM per strand. All NMR spectra were acquired at pH 6.0 and 25 °C. Numbers under signals represent integral values.

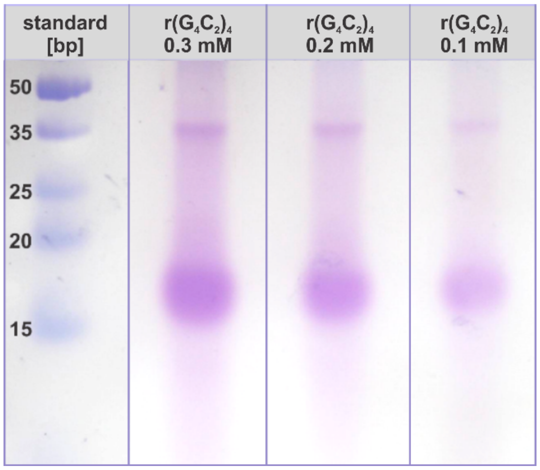

Figure S16. 20% native PAGE of r(G_4C_2)$_4$ at pH 6.0 and 5 °C. Concentration of r(G_4C_2)$_4$ was 0.3, 0.2 and 0.1 mM per strand. DNA ladder was used as a standard.

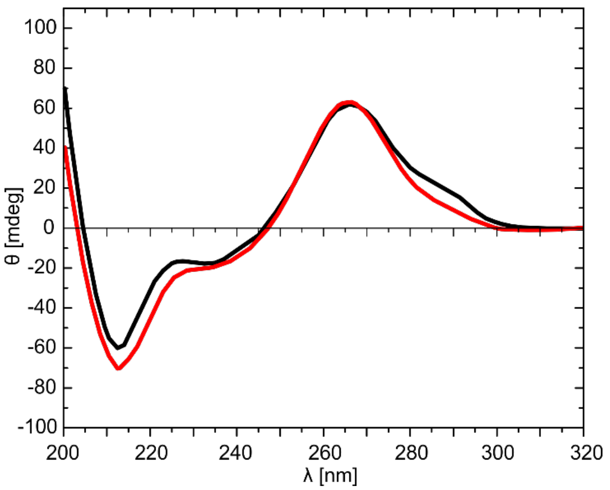

Figure S17. CD spectra of equimolar mixture of r(G_2C_4)$_4$ and r(G_4C_2)$_4$ before (black) and after (red) annealing. Both CD spectra were acquired at pH 6.0, 25 °C and concentration of 100 μM per sense and antisense strand.

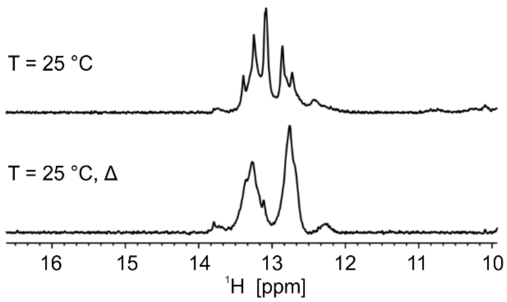

Figure S18. ^{1}H NMR spectra of equimolar mixture of r(G_2C_4)$_4$ and r(G_2C_4)$_4$ before and after annealing. Δ represents NMR spectrum after annealing. Both NMR spectra were acquired at 25 °C, pH 6.0 and concentration of 0.2 mM per sense and antisense strand.

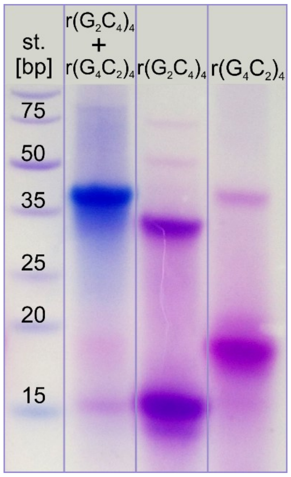

Figure S19. 20% native PAGE of r(G_4C_2)$_4$, r(G_2C_4)$_4$ and r(G_4C_2)$_4$ + r(G_2C_4)$_4$ at pH 6.0 and 5 °C. In all samples concentration of r(G_4C_2)$_4$ and r(G_2C_4)$_4$ was 0.1 mM per strand. DNA ladder was used as a standard.
